# Assessing alternatives to HEPA air purification requirements to reduce viral pathogen transmission in healthcare HVAC systems

**DOI:** 10.1017/ash.2022.217

**Published:** 2022-05-16

**Authors:** Werner Bischoff, David Ornelles, Lauren Ivey, Bill Payne

## Abstract

**Background:** High-efficiency particulate air (HEPA) filters are currently recommended when using recirculated air to eliminate the risk of pathogen transmission such as SARS-CoV-2 from one patient care area to the next. We tested the efficacy of lower-grade air filters in eliminating airborne virus transmission. **Methods:** We conducted an experiment in 2 adjacent exam rooms in an unoccupied hospital emergency unit. The HVAC system contained a 15,000-cubic-feet-per-minute rooftop air handler. All outside air and exhaust dampers were closed during the trial (full air recirculation). We conducted experiments in 3 tests arms with varying grades of MERV filters (AAF Flanders, Louisville, KY): (1) control without filters, (2) MERV8+14 filters, and (3) MERV8+16 filters. We repeated 20-minute virus challenge runs 3 times per test arm. Live attenuated influenza vaccine (2 mL LAIV, FluMist Quadrivalent 2020/21, AstraZeneca, Wilmington, DE), was aerosolized into the HVAC system via a commercial nebulizer. Air was sampled using 3 six-stage Andersen air samplers placed in the center of the adjacent room. Environmental particle counts were collected using a particle counter (PEC-PCO-1, PCE Americas). **Results:** Concentrations of viral RNA were determined by qPCR, and viral concentrations (vg/mL) in each stage of each arm were compared directly. Pairwise comparisons of the virus and particle burdens across each stage of each test arm were made using a general linear model. LAIV was detected in the control arm at a virus burden of 2,277 vg/mL, indicating a >6.5 log reduction of the virus released in the HVAC system (8.8×109 total vg). In the second arm, the MERV8+MERV14 filters demonstrated in a 13-fold decrease in viral burden compared to the control arm (mean virus burden: 169 vg/mL, p Our study demonstrates that viral containing particles can be transported via a hospital HVAC system from one patient room to the next. Considering the decrease in detectable virus within the HVAC system, the combination of MERV8+MERV16 filters reduced the virus burden reaching an adjacent room to levels well below the human infectious dosages for influenza and other highly infective viruses. **Conclusions:** Our findings indicate that MERV8+MERV16 filters provide protection against virus transmission through HVAC systems and are a cost-conscious alternative to HEPA filters.

**Funding:** None

**Disclosures:** None

